# Depressed mood, glycaemic control and functional capacity in overweight/obese men with and without type 2 diabetes

**DOI:** 10.1186/1758-5996-4-46

**Published:** 2012-11-21

**Authors:** Itamar Levinger, Steve Selig, George Jerums, Andrew Stewart, Cadeyrn J Gaskin, David L Hare

**Affiliations:** 1 Institute for Sport, Exercise and Active Living (ISEAL), School of Sport and Exercise Science, Victoria University, PO Box 14428, Melbourne, VIC, 8001, Australia; 2 University of Melbourne and Department of Cardiology, Austin Health, Melbourne, Australia; 3 Centre for Exercise and Sports Science, School of Exercise & Nutrition Sciences, Deakin University, Melbourne, Australia; 4 University of Melbourne and Department of Endocrinology, Austin Health, Melbourne, Australia

**Keywords:** Aerobic power, Cardiac Depression Scale, Depression, Glycaemic control; Type 2 diabetes

## Abstract

**Objective:**

To determine whether there were differences in depressed mood between overweight/obese men with and without type 2 diabetes (T2DM) and to examine any associations between depressed mood, physical functioning, and glycaemic control in overweight/obese men with and without T2DM.

**Methods:**

Fifty seven overweight/obese men with (n = 19, age = 54.2 ± 7.4 yrs, BMI = 32.3 ± 6.7 kg⋅m^-2^) and without T2DM (n = 38, age = 51.1 ± 6.8 yrs, BMI = 29.9 ± 4.5kg⋅m^-2^, p > 0.05 between groups) participated. The men completed measures of depressed mood and health-related quality of life (HRQL) and underwent the following assessments: fasting blood lipids and glucose, HbA1c, anthropometric measurements, VO_2peak_, muscle strength, and physical function.

**Results:**

Compared to men without T2DM, men with T2DM had higher depressed mood (p = 0.05, η^2^ = 0.07), as well as lower perceived general health (p < .01, η^2^ = 0.24) and social functioning (p = .01, η^2^ = 0.10). Men with T2DM also had lower VO_2peak_ (21.8 ± 5.3 versus 25.8 ± 5.4 ml⋅kg^-1^⋅min^-1^, p < .01, η^2^ = 0.11) and muscle strength (3.3 ± 0.8 versus 3.7 ± 0.7 kg⋅kg^-1^, p = 0.08, η^2^ = 0.06), as well as being slower to complete physical performance tasks (27.2 ± 5.2 versus 24.2 ± 2.8 sec, p < 0.01, η^2^ = 0.13). In those with T2DM, depressed mood was highly correlated with most HRQL subscales. For the combined cohort, depressed mood was correlated with fasting glucose (r = 0.31, p = 0.012) but not the functional measures.

**Conclusions:**

Men with T2DM have higher levels of depressed mood compared to men without T2DM. Glycaemic control, but not functional capacities, is associated with depressed mood in the study cohort.

## Introduction

Most people with type 2 diabetes (T2DM) are overweight or obese [1] and, compared to people without diabetes, have double the risk for depression [2]. Aside from experiencing poor health, people with T2DM have higher incidence of functional limitations, particularly if they have poor glycaemic control [3].

The relationship between diabetes and depression is bidirectional [4,5]. Up to a point, the severity of either condition seems to be associated with the chronicity of the other. For example, in community-dwelling older adults, depressive symptoms and HbA1c levels rise in tandem until HbA1c levels reach about 8%, beyond which the severity of both conditions plateau [6]. Although behavioural (e.g. diet, exercise), neurological (activation of the hypothalamic-pituitary-adrenal and sympathoadrenal systems), and pharmacological factors have been suggested to contribute to the incidence of T2DM among people with depression, evidence suggests that the relationship between T2DM and depression is maintained when these factors are taken into account [4]. Similarly, other factors that have been hypothesised to increase the incidence of depressive symptoms among people with T2DM (e.g. presence of comorbidies and diabetic complications) have been shown not to substantially influence the relationship between T2DM and depression [4,7,8]. Further exploration of biological factors may be warranted.

Associations between diabetes and physical function have been established using objective measures (e.g. activities of daily living, ADL) [9]. Being able to maintain an active lifestyle is important for preserving or improving clinical status [10,11] and health related quality of life (HRQL) [12] for people with diabetes. As people with diabetes age, maintenance of ADLs takes on greater significance, because deterioration of ADLs is associated with loss of independence and increased risks of morbidity and mortality [13]. The relationship between physical function and HbA1c levels in people with T2DM is unclear [3,13,14]. Some studies suggest that higher HbA1c levels are associated with impaired physical function [3,13], whilst other research found no evidence of this [14].

Compared to people without T2DM, people with T2DM have lower aerobic capacity [15,16] as well as longer time to recover from exercise [17] and lower muscular strength [18,19]. Maintaining muscular strength is important for reducing the likelihood of functional limitations [20]. Being overweight or obese, as well as having T2DM, may make exercise more challenging, which may contribute further to loss of muscular strength and functional decline, and the cycle repeats itself.

Depression is associated with lower levels of physical [21] and social functioning [22] in people with chronic disease. In people with T2DM, the presence of complications (i.e. diabetic retinopathy, diabetic angiopathy and diabetic neuropathy) has been associated with higher prevalence and severity of both depression and functional disability (indicating lower levels of physical function) [23]. Further, people with poor self-perceived weight control had more depressive symptoms and lower physical functioning, and that self-perceived weight control was linearly related to BMI [23].

People with diabetes and co-morbid depression experience diminished HRQL [24,25]. Although relationships are thought to exist between depression and mental health components of HRQL in people with diabetes, the relationship between depression and physical functioning is not clear [24].

Evidence suggests that men and women may experience different levels of depression associated with T2DM [26]. In this study, we focused on the experiences of middle-aged men to exclude the potential effects of menopause (metabolism and psychological). The aims of the current study were (a) to determine whether there were differences in depressed mood between overweight/obese men with and without T2DM and (b) to examine the associations between depressed mood, functional measures and glycaemic control in men with and without T2DM. We hypothesized that patients with T2DM have higher depressed mood and that depressed mood would be correlated with impairment of physical function and poorer glycaemic control.

## Methods

### Participants

Inclusion criteria: males with and without T2DM ≥40 yr not participating in regular aerobic or resistance exercise for the previous six months; Exclusion criteria: symptomatic cardiovascular disease or musculoskeletal conditions that impaired physical activity. Participants were overweight or obese men with (n = 19) or without (n = 38) T2DM (Table 1). Participants were on a range of medications (Table 1).

**Table 1:** Comparison between overweight/obese men without diabetes (n = 38) and overweight/obese men with diabetes (n = 19) on demographics and medications

	**Men without Diabetes**	**Men with Diabetes**	**η**^**2**^	**p**
Demographics	-------	-------	-------	-------
Age (yrs)	51.1 ± 6.8	54.2 ± 7.4	.04	.12
Height (cm)	175.9 ± 6.7	175.9 ± 5.8	.00	.99
Mass (kg)	92.4 ± 14.0	100.2 ± 23.0	.04	.12
BMI (kg⋅m-2)	29.9 ± 4.5	32.3 ± 6.7	.04	.12
Fasting Glucose (mmol · L-1)	5.4 ± 0.5	8.1 ± 1.9	.54	<.01*
HbA1c (%)	5.5 ± 0.3	6.8 ± 0.9	.51	<.01*
HbA1c (mmol · mol-1)	37 ± 4	50 ± 10	.51	<.01*
Trig (mmol · L-1)	1.5 ± 0.9	1.7 ± 0.8	.02	.35
HDL (mmol · L-1)	1.2 ± 0.4	1.0 ± 0.2	.08	.04*
SBP (mmHg)	130.0 ± 14.3	133.7 ± 16.4	.01	.38
DBP (mmHg)	86.6 ± 11.0	86.2 ± 13.3	.00	.90
Known duration of T2DM (yrs)	-------	2.5 ± 3.6	-------	-------
Medications	-------	-------	-------	-------
Calcium channel blockers	0	3	-------	-------
ACE inhibitors	5	4	-------	-------
Beta-blockers	2	3	-------	-------
Cholesterol lowering medication	2	11	-------	-------
Glucose lowering medication	0	12	-------	-------
Angiotensin II receptor antagonist	1	4	-------	-------
Platelet inhibitor	0	2	-------	-------
Anti-depressants	2	3	-------	-------
Aspirin	0	6	-------	-------

*p < .05. *BMI* (body mass index), *HbA1c* (glycosylated-haemoglobin), *Trig* (triglyceride), *HDL* (high density lipoprotein), *SBP* (systolic blood pressure), *DBP* (diastolic blood pressure). Data are mean  ±  SD.

The Victoria University and Austin Health Human Research Ethics Committees approved the study protocol. The participants provided informed consent before becoming involved with the study.

### Measures

#### Demographics

Participants were asked to provide their ages, what medications they were taking, and for those with T2DM, the known duration of the disease.

#### Blood measures

A blood sample was collected after overnight fasting. Blood was analysed (SYNCHRON LX® System/Lxi725, Beckman Coulter Inc, CA, USA) for triglyceride (Trig), high-density lipoprotein (HDL), glucose, and HbA1c.

#### Blood pressure

Blood pressure was measured using a mercury sphygmomanometer, as has been described previously [27].

#### Anthropometric measurements

Height was measured with participants standing barefoot on a stadiometer (±0.5 cm). Weight was measured with participants wearing just underwear while standing on a calibrated scale (August Sauter GmbH, Germany) (±0.05 kg).

#### Aerobic power

The full protocol for measuring aerobic power has been described previously [27]. In brief, aerobic power (VO_2peak_) was assessed using a sign and symptom limited graded exercise test on Cybex MET 100 cycle (Cybex Metabolic Systems, Ronkonkoma, NY, USA). VO_2_ for each 15 sec interval was measured by gas analysis (Medgraphics, Cardio2 and CPX/D System with Breezeex Software, 142090–001, Revia, MN, USA).

#### Physical function

The Physical Performance Test (PPT) was used as an objective measure of physical function. The full protocol of the PPT has been described previously [27]. In brief, the PPT included four functional mobility tasks, including a 15 meters rapid walking test; a timed up-and-go test where participants were required to rise from a standard chair, walk 3 meters, and return to a seated position on the chair; and stair climbing and stair descending tests. All tests were timed (sec). The PPT score was the sum of the fastest times for each of the four tests.

#### Muscular strength

Muscular strength was evaluated using the one repetition maximum method for chest press and leg press, as described previously [27]. Muscle strength was calculated as the sum of weight lifted in the two exercises, and is expressed as relative to body mass.

#### Depressed mood

The Cardiac Depression Scale (CDS) was used as a measure of depressed mood in this populations as many people with T2DM also experience co-morbid cardiovascular disease or are at a high risk of developing cardiovascular disease The CDS contains 26 items, to which participants responded on a 7-point Likert scale [28]. Higher scores are indicative of more severely depressed mood. Scores ≥95 may be indicative of major depression [29]. The CDS is a reliable tool to assess depressive symptoms in people at a high risk of developing T2DM with a Cronbach’s alpha =0.84 and is sensitive for changes in depressive symptoms post-exercise training [30].

#### Health-related quality of life

The SF-36 [31] was used to measure HRQL. This instrument has eight subscales: physical functioning, role limitations-physical, role limitations-emotional, energy/fatigue, emotional well-being, social functioning, pain, and general health. The SF36 is well accepted and one of the most used questionnaires to assess HRQL in many clinical and healthy populations

### Statistical analyses

Where appropriate, data are reported as mean ± standard deviation (SD). An analysis of variance (ANOVA) was used to determine whether there were differences between men with and without T2DM with respect to their scores on the measures of physical function, HRQL, depressed mood, aerobic power, and muscular strength. In addition to statistical significance, effect sizes for the difference between the two conditions, in the form of η^2^, were calculated. η^2^ values provide the proportion of variability in a dependent variable that can be explained by the independent variable. Although η^2^ values for differences on physical measures vary widely (i.e., between 0 and 1), on social and psychological measures (e.g., depression, HRQL) small, medium, and large effects are 0.01, 0.06, and 0.14, respectively [32]. Pearson’s product moment correlations were calculated to investigate the relationships between depressed mood and functional measures, HRQL, and glycaemic control. Although correlation coefficients (r) can vary widely when physical measures are being correlated (between −1 and 1), on social and psychological measures small, medium, and large effects are 0.1, 0.3, and 0.5, respectively [32].

All statistical analyses were conducted at the 95% level of significance and data reported as mean ± standard deviation (SD).

## Results

Men with T2DM had higher fasting glucose and HbA1c levels, and slightly lower HDL levels, compared to those without T2DM (Table 1). There were no significant differences between men with and without T2DM; however, with respect to age, height, mass, BMI, triglycerides, and systolic and diastolic blood pressure. Men with T2DM performed the physical performance tasks more slowly, reported poorer perceptions of their general health and social functioning and higher levels of depressed mood, and recorded lower aerobic power (Table 2). Depressed mood was correlated with several aspects of HRQL (general health, energy/fatigue, and emotional well-being) in men with and without T2DM (Table 3). For each group separately there were no significant correlations between depressed mood and physical function, aerobic power, muscular strength, and glycaemic control. However, in the entire cohort (pooled data), depressed mood correlated with fasting glucose (r = 0.31, p = 0.012) but not aerobic power, muscle strength or the capacity to perform ADLs (data adjusted for BMI). There was no correlation between depressed mood and BMI (adjusted and not adjusted for fasting glucose).

**Table 2:** Comparison between overweight/obese men without diabetes (n = 38) and overweight/obese men with diabetes (n = 19) on physical function, health-related quality of life, depressed mood, aerobic capacity, and muscular strength

	**Men without Diabetes**	**Men with Diabetes**	**η**^**2**^	**p**
Physical Function	-------	-------	-------	-------
Physical performance (secs)	24.2 ± 2.8	27.3 ± 5.2	.13	<.01*
Health-Related Quality of Life	-------	-------	-------	-------
Physical functioning	87.6 ± 12.0	82.1 ± 12.4	.05	.11
Role limitations – physical	81.6 ± 32.2	76.3 ± 33.8	.01	.57
Role limitations – emotional	88.6 ± 27.2	82.4 ± 35.8	.01	.47
Energy/fatigue	62.4 ± 14.5	60.8 ± 14.5	.00	.70
Emotional well-being	79.5 ± 11.9	76.0 ± 13.5	.02	.32
Social functioning	91.6 ± 13.9	79.7 ± 21.3	.10	.01*
Pain	72.9 ± 21.9	80.3 ± 18.0	.03	.21
General health	69.1 ± 17.0	49.3 ± 16.9	.24	<.01*
Depressed mood	72.3 ± 20.1	83.9 ± 21.1	.07	.05
Aerobic Power (VO_2peak_)	25.8 ± 5.4	21.8 ± 5.3	.11	<.01*
Muscular Strength (kg · kg)	3.7 ± 0.7	3.3 ± .8	.06	.08

*p < .05. Data are mean  ±  SD.

**Table 3:** Relationships between depressed mood and physical function, health-related quality of life, aerobic power, muscular strength, and glycaemic control in overweight and obese men with and without T2DM

	**Depressed Mood in Men without Diabetes**		**Depressed Mood in Men with Diabetes**	
	**r**	**p**	**r**	**p**
Physical Function	-------	-------	-------	-------
Physical performance	.15	.18	-.07	.39
Health-Related Quality of Life	-------	-------	-------	-------
Physical functioning	-.40	<.01*	-.35	.07
Role limitations – physical	-.47	<.01*	-.58	<.01*
Role limitations – emotional	-.16	.17	-.54	<.01*
Energy/fatigue	-.67	<.01*	-.85	<.01*
Emotional well-being	-.67	<.01*	-.56	<.01*
Social functioning	-.31	.03	-.80	<.01*
Pain	-.38	<.01*	-.67	<.01*
General health	-.70	<.01*	-.34	.08
Aerobic Power	-.17	.16	.26	.15
Muscular Strength	-.23	.08	.20	.21
Glycaemic Control	.08	.33	-.04	.44

*p < .05.

## Discussion

This study showed that men with T2DM have higher levels of depressed mood than those without T2DM. T2DM explained 7% of the variance in depressed mood scores, which is a moderately large effect for a psychological measure [32]. This finding supports previous work [2,33] and suggests that clinicians need to be vigilant for the presence of co-morbid symptoms of depression when treating men with T2DM. This is supported by evidence that the relationship between diabetes and depression is bidirectional [4,5].

Consistent with previous work that has evaluated aerobic capacity [15,16,19] and strength [18,19] for people with T2DM, the present study showed that men with T2DM had lower aerobic capacity and strength than men without T2DM. In addition, men with T2DM had lower physical function scores (both objective measures and self-perceived). Taken together, these findings emphasise the value for people with T2DM to undertake regular physical activity.

In both men with and without T2DM, depressed mood was strongly associated with subjective measures of functioning (subscales of HRQL) but weakly related to objective measures of functioning (physical performance, aerobic power, muscular strength). This finding may mean that men with depressed mood may perceive their physical functioning to be worse than is evidenced through objective measures, particularly those with T2DM. Such perceptions may lead to lower self-efficacy and discourage people with T2DM from undertaking regular exercise and physical activity that they are capable of performing, which may, in turn, have detrimental effects on clinical, functional and psychosocial wellbeing. For this reason, an interventional study is warranted for people with T2DM that aims to relieve symptoms of depression and improve self-efficacy for exercise; outcome measures could include effects on long term exercise participation, physical functioning and HRQL.

Depressed mood was strongly correlated with social functioning in men with T2DM. The relationship between depression and social function is often significant and pervasive, but is frequently underappreciated [34]. As part of treatments for both T2DM and depression, clinicians should enquire about the social lives of men with diabetes and work with them to build on their relationships with others.

In men with diabetes, a large correlation was also found between depressed mood and pain. Although causation cannot be determined from this study, it seems likely that pain could exacerbate depressed mood as it may limit a person’s physical functioning and willingness to exercise. With at least one quarter of people with diabetes affected by distal symmetric polyneuropathy [35], with which people often experience debilitating neuropathic pain, clinicians need to account for pain as a cause for depressed mood in their patients.

There is evidence that for people with T2DM, depression is associated with increased insulin resistance and poor glycaemic control, whilst the links between glycaemic control and functional capacities are weaker for people with T2DM [36]. The current study supports this finding. Fasting glucose, but not objective measures of physical function, was correlated with depressed mood. This may have important implications for the prevention, and management of depression in this population. It highlights that improvements in glycaemic control may not only have significant clinical benefits, such as reductions in diabetic complications [37], but may also have psychological benefits, and *viceversa*. The current study has several potential limitations including the cross-sectional nature of the study, a relative small sample size and that these findings may not generalize to women or other demographic groups.

In conclusion, men with T2DM have higher levels of depressed mood and lower functional capacity, as measured by both objective and subjective tools, compared to men without diabetes. Increased fasting blood glucose levels are associated with increased depressive symptoms.

## Competing interests

The authors declare that they have no conflict of interest.

## Authors’ contribution

IL participated in the concept and design, data collection, analysis, interpretation of the results and drafting the paper. SS participated in the concept and design, interpretation of the results, review of the paper and supervision. GJ participated in the concept and design, interpretation of the results and critical review of the paper. AS participated in data interpretation and drafting the paper. CJG participated in data analysis, interpretation of the results and drafting the paper. DLH participated in the concept and design, interpretation of the results and critical review of the paper. All authors read and approved the final manuscript.
